# Opposite Effects of Neuropeptide FF on Central Antinociception Induced by Endomorphin-1 and Endomorphin-2 in Mice

**DOI:** 10.1371/journal.pone.0103773

**Published:** 2014-08-04

**Authors:** Zi-long Wang, Quan Fang, Zheng-lan Han, Jia-xin Pan, Xu-hui Li, Ning Li, Hong-hai Tang, Pei Wang, Ting Zheng, Xue-mei Chang, Rui Wang

**Affiliations:** Key Laboratory of Preclinical Study for New Drugs of Gansu Province, and Institute of Physiology, School of Basic Medical Sciences, Lanzhou University, Lanzhou, PR China; University of Lübeck, Germany

## Abstract

Neuropeptide FF (NPFF) is known to be an endogenous opioid-modulating peptide. Nevertheless, very few researches focused on the interaction between NPFF and endogenous opioid peptides. In the present study, we have investigated the effects of NPFF system on the supraspinal antinociceptive effects induced by the endogenous µ-opioid receptor agonists, endomorphin-1 (EM-1) and endomorphin-2 (EM-2). In the mouse tail-flick assay, intracerebroventricular injection of EM-1 induced antinociception via µ-opioid receptor while the antinociception of intracerebroventricular injected EM-2 was mediated by both µ- and κ-opioid receptors. In addition, central administration of NPFF significantly reduced EM-1-induced central antinociception, but enhanced EM-2-induced central antinociception. The results using the selective NPFF_1_ and NPFF_2_ receptor agonists indicated that the EM-1-modulating action of NPFF was mainly mediated by NPFF_2_ receptor, while NPFF potentiated EM-2-induecd antinociception via both NPFF_1_ and NPFF_2_ receptors. To further investigate the roles of µ- and κ-opioid systems in the opposite effects of NPFF on central antinociception of endomprphins, the µ- and κ-opioid receptors selective agonists DAMGO and U69593, respectively, were used. Our results showed that NPFF could reduce the central antinociception of DAMGO via NPFF_2_ receptor and enhance the central antinociception of U69593 via both NPFF_1_ and NPFF_2_ receptors. Taken together, our data demonstrate that NPFF exerts opposite effects on central antinociception of endomorphins and provide the first evidence that NPFF potentiate antinociception of EM-2, which might result from the interaction between NPFF and κ-opioid systems.

## Introduction

In 1985, NPFF was isolated from bovine medulla oblongata and characterized as an anti-opioid peptide [1]. In previous studies, the NPFF system was found including two precursors (pro-NPFF_A_ and pro-NPFF_B_) and two G-protein-coupled receptors (NPFF_1_ and NPFF_2_) [2]–[7]. Moreover, the pro-NPFF_A_ peptides (such as NPA-NPFF) and pro-NPFF_B_ peptides (such as NPVF) were suggested to be the preferred ligands for NPFF_2_ and NPFF_1_ receptors, respectively [5], [8]. Additionally, in the structure-activities studies and pharmacological assays, NPVF and dNPA were demonstrated to be the most selective agonists of NPFF_1_ and NPFF_2_ receptors, respectively [5], [8]–[10].

NPFF system was demonstrated to mediate a variety of biological actions, such as food intake, body temperature, gastrointestinal modulation, cardiovascular and nociceptive action [11]–[13]. In addition, NPFF and opioid systems had been shown to interact at several levels, from receptor molecules to animal behavior [11], [14]. At the cellular level, NPFF and related peptides were found to exhibit anti-opioid effects via NPFF_1_ and NPFF_2_ receptors in isolated neurons and recombinant cellular models [15]–[20]. However, at the whole animal level, the opioid-modulating activities of NPFF vary in different pharmacological studies [12], [14]. Intracerebroventricular (i.c.v.) administration of NPFF and related peptides exerted opioid-like inhibition of small intestinal transit and delayed colonic bead propulsion in mice [21], [22]. In contrast, the previous reports suggested that supraspinal administration of NPFF exerted anti-opioid properties in feeding behaviour, locomotor activity and rewarding effect [11], [14]. NPFF produced a bimodal effect on pain perception: NPFF receptors agonists exerted anti- or pro-opioid effects depending on their route of administration and the level of opioid-induced analgesia [13], [23]. In general, supraspinal administrations of NPFF and related peptides were found to play an anti-opioid role. Intracerebroventricular administration of NPFF attenuated the analgesic effect of morphine [1], [24], [25]. In contrast, intrathecal administration of NPFF exerted a pro-opioid role and induced an opioid-like analgesia or potentiated morphine analgesia [26], [27].

To date, the link between NPFF and morphine has been widely investigated [11], [14]. However, very few researches focused on the modulating effects of NPFF on the endogenous opioid peptides. In fact, unlike morphine, the endogenous opioid peptides have different mechanisms in pain modulation. In previous studies, two endogenous opioid tetrapeptides, endomorphin-1 (EM-1) and endomorphin-2 (EM-2) have been shown to activate µ-opioid receptor with high affinity but have no appreciable affinity with δ-opioid receptor and κ-opioid receptor [28]–[30]. Many studies have shown that endomorphins functioned as two selective endogenous µ-opioid receptor ligands [28], [29], [31]. Although EM-1 and EM-2-induced antinociception were both mediated by the activation of µ-opioid receptors, they likely produced their analgesic effects via different mechanisms [32]. EM-1-induced antinociception was mediated by µ-opioid receptor similar to morphine or DAMGO. However, EM-2 initially stimulated µ-opioid receptor, which subsequently induced the release of dynorphins that act on κ-opioid receptor to produce antinociception [32]–[34].

In the present study, the effects of NPFF and related peptides on the supraspinal antinociception of EM-1 and EM-2 were investigated in the mouse tail-flick assay. Our results demonstrated that central administration of NPFF significantly reduced the antinociception of EM-1, but enhanced EM-2-induced antinociception. In addition, the enhancement of NPFF on EM-2-induced antinocicetion might be related to the modulating effect of NPFF on κ-opioid receptor at the supraspinal level.

## Materials and Methods

### Animals

All the experiments were conducted on the male Kunming mice, which were provided by the Experimental Animal Center of Lanzhou University. All animals were cared for and experiments were carried out in accordance with the European Community guidelines for the use of experimental animals (86/609/EEC). Animals were housed in an animal room that was maintained at 22±2°C with a 12-h light/12-h dark cycle and given free access to food and water. All the protocols in this study were approved by the Ethics Committee of Lanzhou University (permit number: SYXK Gan 2009-0005), China.

### Chemicals

EM-1 (YPWFamide), EM-2 (YPFFamide), NPFF (FLFQPQRFamide), NPVF (VPNLPQRFamide), dNPA (*D*.NP(*N*-Me)AFLFQPQRFamide) and RF9 were synthesized by manual solid-phase synthesis using standard N-fluorenylmethoxycarbonyl (Fmoc) chemistry following the previous report. [35] The crude peptides were firstly desalted by Gel filtration, and then purified by preparative RP-HPLC using a Waters Delta 600 system coupled to a UV detector. Analytical RP-HPLC was used to establish the purity of those peptides. The molecular weights of the peptides were confirmed by an electrospray ionization mass spectrometer (Mariner ESI-TOF MS, Applied Biosystems, CA).

In addition, the µ-opioid receptor selective agonist DAMGO ([D-Ala^2^, N-MePhe^4^, Gly-ol]-enkephalin), κ-opioid receptor selective agonist U69593 ([5a,7a,8b]-(+)-*N*-methyl-*N*-[7-(1-pyrrolidinyl)-1-oxaspiro[4.5]dec-8-yl] benzeneacetamide) and µ-, κ- and δ-opioid receptors selective antagonists beta-funaltrexamine (β-FNA), nor-binaltorphimine (nor-BNI) and naltrindole (NTI) respectively, were purchased from Sigma Chemical Company (USA). All drugs were dissolved in sterilized saline, and stored in 1.5 ml tubes at –20°C.

### Implantation of cannula into lateral ventricle

The method has been described in our previous study [36]. Briefly, mice (18–20 g) were anesthetized with pentobarbital sodium (80 mg/kg, intraperitoneally) and placed in a stereotaxic apparatus. A sagittal incision was made in the midline exposing the surface of the skull, and a stainless steel guide cannula was implanted into the left or right lateral ventricle. The coordinates are 3.0 mm posterior and 1.0 mm lateral to the bregma and 3.0 mm ventrally from the surface of skull. The guide cannula was fixed with dental cement. A dummy cannula was inserted into the guide cannula to block the passage that the cannula is not in use. After surgery, the animals were housed individually and allowed to recover for at least 4 days.

### Administration of drugs

Drugs were injected i.c.v. through the implanted cannula. Each mouse was injected in a volume of 4 µl at a period of 30 seconds using a 25-µl microsyringe, followed 1 µl of saline to flush the catheter. After completion of behavioral testing, i.c.v. administration of methylene blue dye was used to verify the proper injection site. Only the data from those animals with dispersion of the dye throughout the ventricles were used in the study.

### Nociceptive test

The nociceptive response was assessed by the radiant heat tail-flick test. Briefly, the animals were gently restrained by hand, and place the underside of the tail 3 cm from its distal end on the radiant heat source. The time of mouse flick its tail off the heat source is defined as the tail-flick latency. The radiant heat intensity that produced a baseline response within 3–5 s was selected in the experiments. A cut-off time was set at 10 s to avoid tissue damage. Tail-flick latency was determined before injection and then at 5, 10, 15, 20, 30, 45 and 60 min after injection. Data are expressed as the maximum possible effect (MPE) calculated as: MPE (%) = 100×[(post-drug response–baseline response)/(cut-off response–baseline response)]. The raw data from each animal were converted to area under the curve (AUC). We calculated the AUC data over the period 0 to 30 or 60 min which were used to statistically analysis.

### Experimental design

In the present study, experiments were designed to examine the effects of the NPFF system on the central antinociception induced by EM-1 and EM-2. Firstly, to investigate which opioid receptors were involved in the central antinociception of EM-1 and EM-2, the selective antagonists β-FNA, nor-BNI and naltrindole were injected 4 h, 30 min, 20 min, respectively, prior to endomorphins. Secondly, in order to investigate the effects of NPFF on EM-1 and EM-2-induced central antinociception, NPFF was injected alone or co-injected with the antagonist RF9 [37], [38] by the i.c.v. route 20 min prior to endomorphins. Moreover, to further investigate the role of NPFF receptor subtypes in modulatory effects of EM-1 and EM-2-induced central antinociception, the NPFF_1_ and NPFF_2_ receptors selective agonists, NPVF and dNPA were used in the present study. NPVF and dNPA were also injected alone or co-injected with RF9 by the i.c.v. route 20 min prior to endomorphins. Lastly, to further investigate the mechanism of NPFF-induced the opposite effects on central antinociception of endomorphins, the µ-opioid receptor selective agonist DAMGO and κ- opioid receptor selective agonist U69593 were used to investigate the interaction between NPFF and µ- or κ-opioid system. Thus NPFF and related peptides were injected alone or co-injected with RF9 by the i.c.v. route 20 min prior to DAMGO or U69593.

### Statistical analysis

All experiments were separately conducted on 7–8 mice. Data were expressed as means ± S.E.M. The significance between groups was analyzed with one-way ANOVA followed by Bonferroni’s post hoc test. Probabilities of less than 5% (P<0.05) were considered as statistical significance. The dose that elicits 50% efficacy (EC_50_) at peak effect and the corresponding 95% confidence limits were determined using Graphpad Prism 5 (Graphpad Software, Inc., La Jolla, CA).

## Results

### Effects of i.c.v. administration of β-FNA, nor-BNI and naltrindole on the central antinociception induced by EM-1 or EM-2

**Figure 1:**
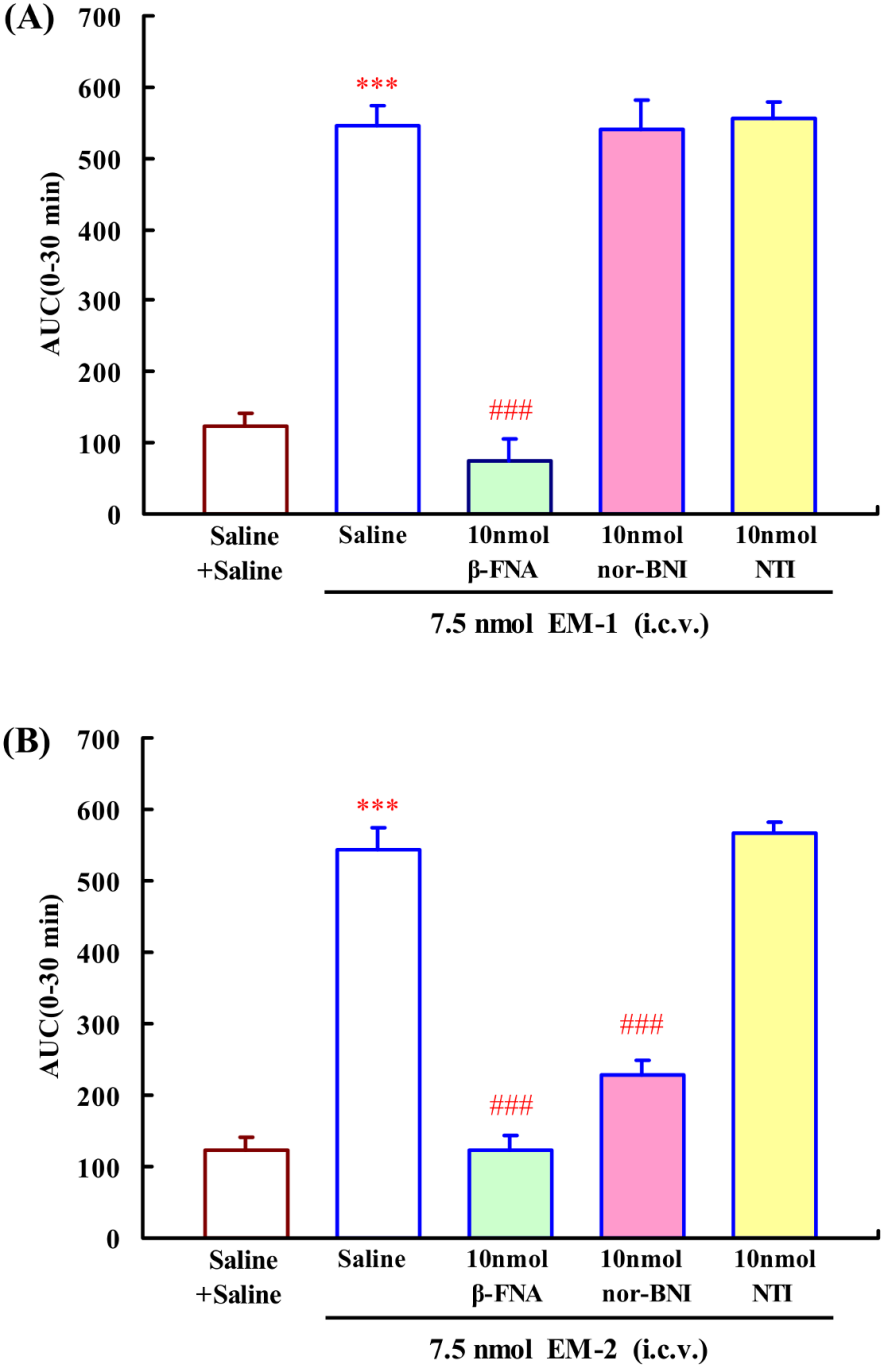
Effects of i.c.v. administration of β-FNA (10 nmol), nor-BNI (10 nmol) and naltrindole (10 nmol) on the EM-1 (7.5 nmol i.c.v.) (A) or EM-2 (7.5 nmol i.c.v.) (B) induced central antinociception in mouse tail-flick test. Data are expressed as differences in AUC between endomorphins (7.5 nmol) and endomorphins co-injected with β-FNA, nor-BNI or NTI during 0–30 min. Each value represents mean ± S.E.M. (n = 7–12 mice/group). ***p<0.001 indicating significant differences compared to Saline + Saline-injected group, ^###^p<0.001 indicating significant differences compared to Saline + endomorphins-injected group.

In the mouse tail-flick test, compared to saline group, i.c.v. injection of 7.5 nmol EM-1 or EM-2 produced significant increases in tail withdrawal latencies (*P*<0.001) (Fig. 1). The opioid receptors selective antagonist β-FNA, nor-BNI and naltrindole were used to further investigate the mechanism of EM-1 and EM-2-induced central antinociception. As show in Fig. 1A, pretreatment with µ-opioid receptor selective antagonist β-FNA completely blocked the EM-1-induced central antinociception, *F*_4,31_ = 69.519, *P*<0.001. However, neither nor-BNI nor naltrindole altered the central antinociception of EM-1 (*P*>0.05). In contrast, pretreatment with β-FNA completely blocked the EM-2-induced central antinociception and κ-opioid receptor selective antagonist nor-BNI partially but significantly blocked the EM-2-induced central antinociception, *F*_4,35_ = 66.967, *P*<0.001. However, δ-opioid receptor selective antagonist naltrindole had no effect on EM-2-induced central antinociception (*P*>0.05) (Fig. 1B).

### Effects of i.c.v. administration of NPFF on the central antinociception produced by EM-1 or EM-2

**Figure 2:**
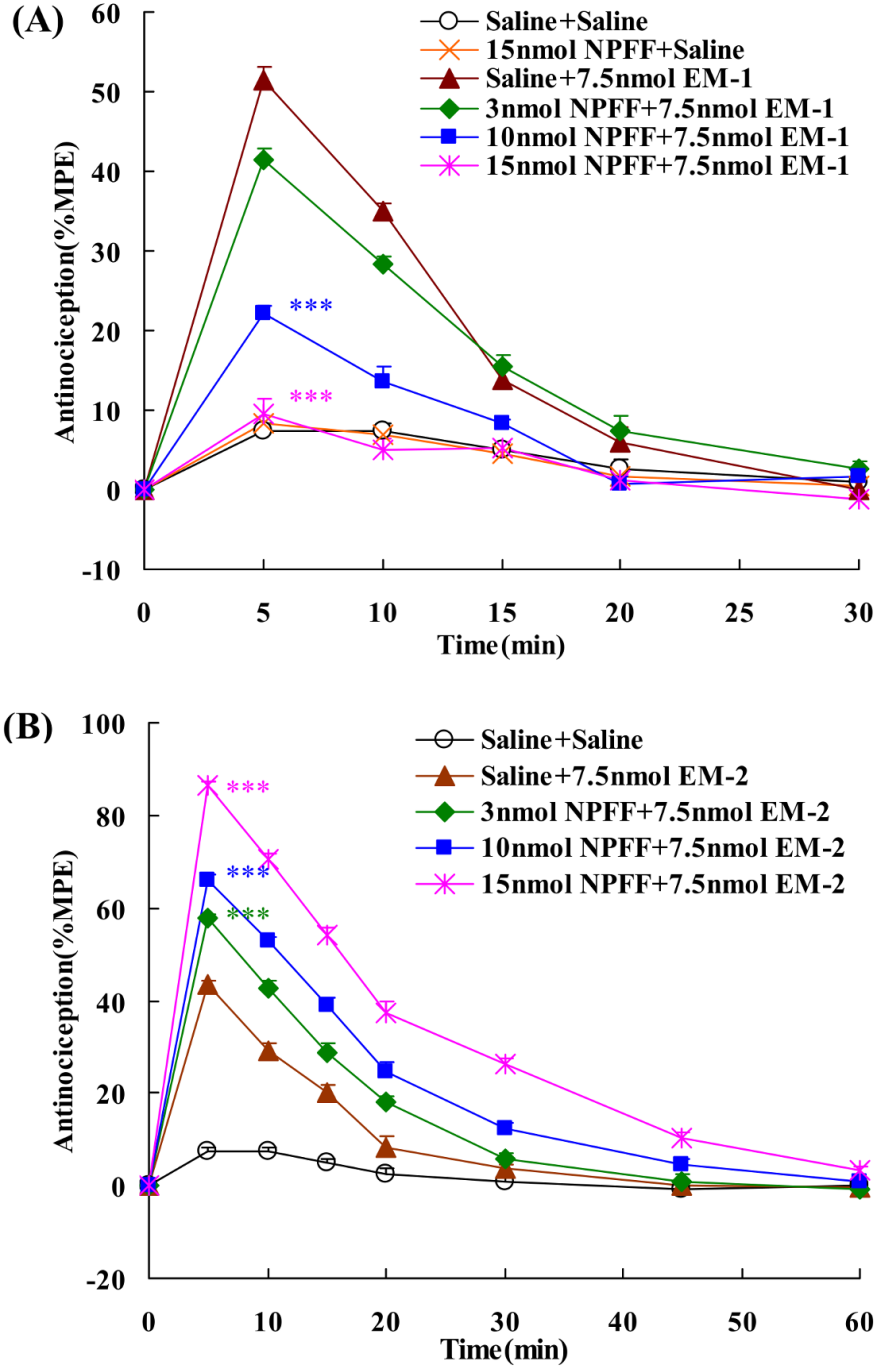
Dose-related effects of i.c.v. administration of NPFF on the central antinociception of EM-1 (i.c.v.) (A) and EM-2 (i.c.v.) (B), in mouse tail-flick assays. (A) NPFF (3, 10 and 15 nmol) reduced 7.5 nmol EM-1-induced central antinociception. (B) NPFF (3, 10 and 15 nmol) potentiated 7.5 nmol EM-2-induced central antinociception. Each value represents mean ± S.E.M. (n = 7–12 mice/group). ***p<0.001 indicating significant differences compared to Saline + endomorphins - injected group.

A dose of 7.5 nmol EM-1 or EM-2 injected i.c.v. which induced 51% or 43% analgesia, respectively, was chosen to investigate both potentiation and reversion of the central antinociception (Fig. 2). Intracerebroventricular administration of NPFF had no significant effect on nociceptive threshold, but dose-dependently reduced the central antinociception of EM-1 with an EC_50_ value (and 95% confidence limits) of 5.92 (5.04–6.96) nmol, *F*_4,34_ = 106.246, *P*<0.001 (Fig. 2A). In contrast, i.c.v. administration of NPFF (3, 10, 15 nmol) markedly evoked significant increases of the central antinociception induced by EM-2, *F*_4,38_ = 281.514, *P*<0.001, the EC_50_ value (and 95% confidence limits) is 9.04 (7.42–11.01) nmol (Fig. 2B).

**Figure 3:**
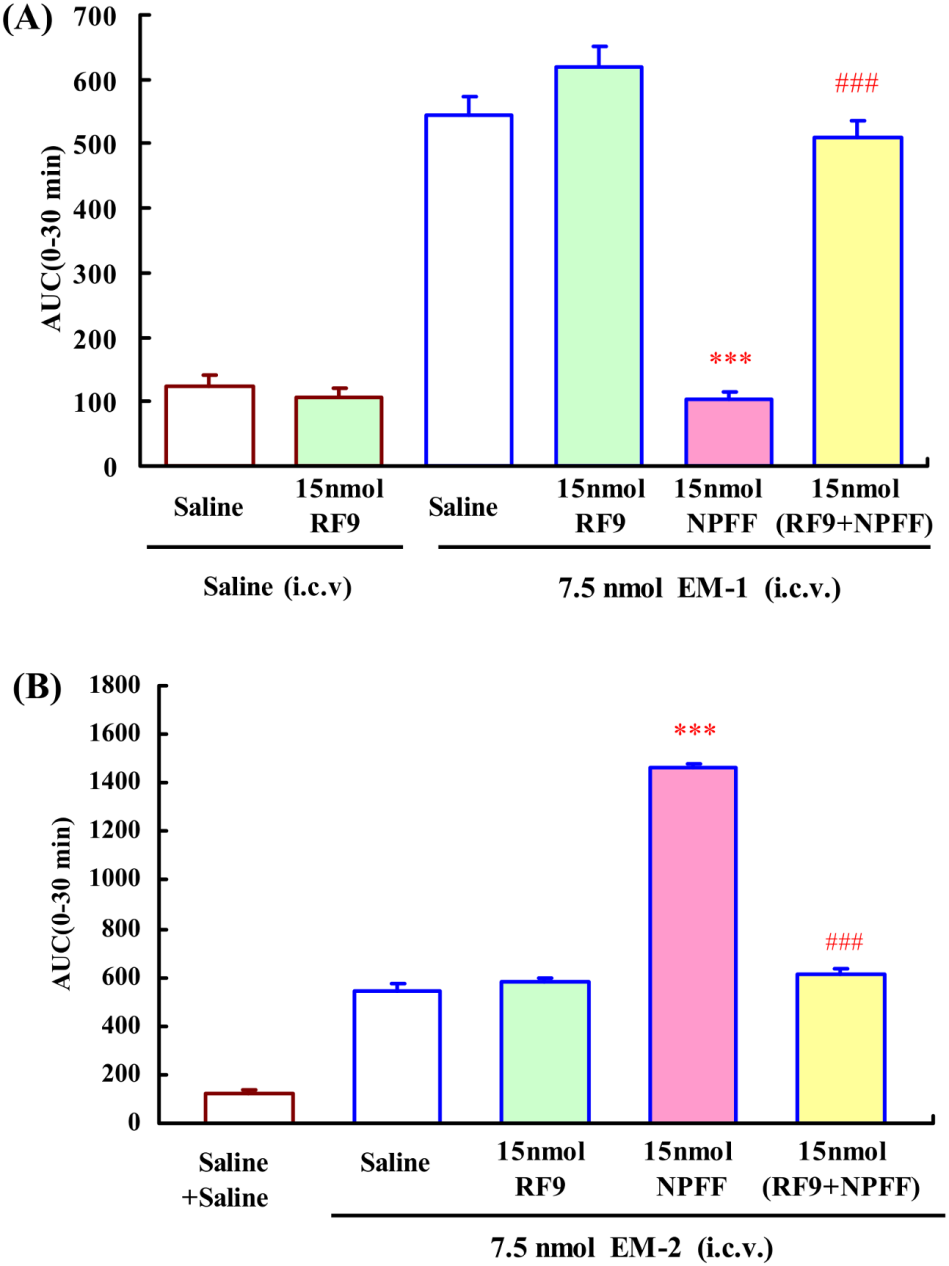
Co-administrated RF9 (15 nmol, i.c.v.) antagonized the modulatory effects of NPFF (15 nmol, i.c.v.) on EM-1 and EM-2-induced central antinociception in mouse tail-flick test. Data are expressed as differences in AUC between endomorphins (7.5 nmol) and endomorphins co-injected with NPFF, RF9 or agonists plus RF9 during 0–30 min. Each value represents mean ± S.E.M. (n = 7–8 mice/group). ***P<0.001 indicating significant differences compared to Saline + endomorphins - injected group; ^###^P<0.001 indicating significant differences from the modulatory effects of NPFF in the absence of RF9.

Furthermore, the NPFF receptors antagonist RF9 was co-injected with NPFF to explore whether the NPFF receptors are involved in the modulatory activities of NPFF. The results showed that i.c.v. RF9 (15 nmol, i.c.v.) itself had no effect on the nociceptive threshold and the central antinociception induced by EM-1 or EM-2. While 15 nmol RF9 (i.c.v.) completely blocked the modulating effects of NPFF on EM-1 and EM-2-induced central antinociception in mice, *F*_3,27_ = 121.470, *P*<0.001; *F*_3,31_ = 359.075, *P*<0.001, respectively (Fig. 3).

### Effects of i.c.v. administration of NPVF and dNPA on the central antinociception produced by EM-1 or EM-2

**Figure 4:**
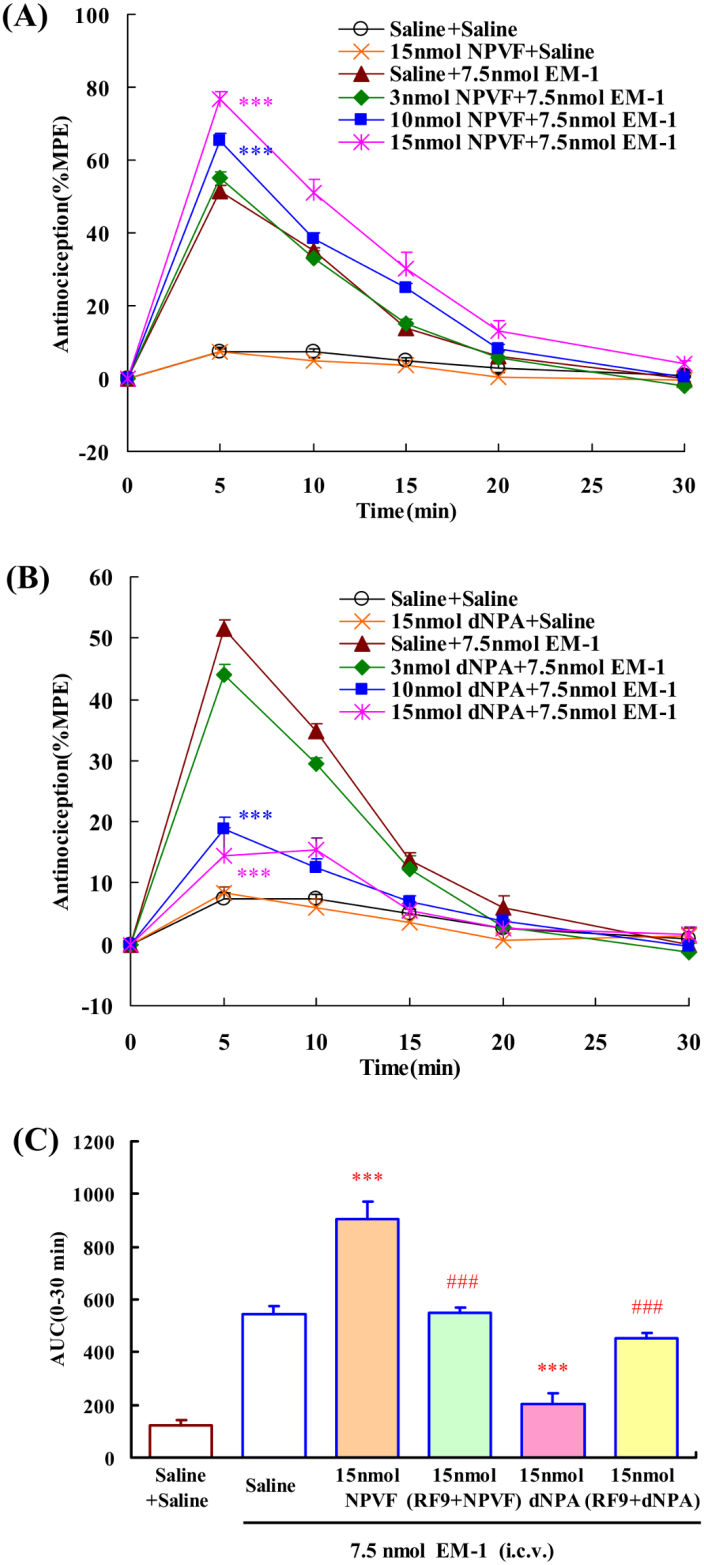
Dose-related effects of i.c.v. administration of NPFF receptors selective agonists on EM-1 (i.c.v.) induced central antinociception in mouse tail-flick test. (A) NPVF (3, 10 and 15 nmol) potentiated EM-1(7.5 nmol) induced central antinociception. (B) dNPA (3, 10 and 15 nmol) reduced EM-1 (7.5 nmol) induced central antinociception. (C) Co-administration of RF9 (15 nmol, i.c.v.) antagonized the effects of NPFF (15 nmol, i.c.v.) on EM-1-induced central antinociception in mouse tail-flick test. Each value represents mean ± S.E.M. (n = 7–8 mice/group). ***P<0.001 indicating significant differences compared to Saline + EM-1-injected group. ^###^P<0.001 indicating significant differences from the modulatory effects of NPFF in the absence of RF9.

To investigate the roles of NPFF receptor subtypes in the modulating effects of EM-1 and EM-2-induced central antinociception, the NPFF_1_ and NPFF_2_ receptors selective agonists, NPVF and dNPA were used, respectively. The effects of NPVF and dNPA on EM-1-induced central antinociception were shown in Fig. 4. I.c.v. administration of NPVF or dNPA had no significant effect on the nociceptive threshold. I.c.v. administration of NPVF (3, 10, 15 nmol) dose-dependently enhanced the central antinociception of EM-1 with an EC_50_ value (and 95% confidence limits) of 14.98 (12.79–17.56) nmol, *F*_4,33_ = 63.812, *P*<0.001 (Fig. 4A). In contrast, i.c.v. administration of dNPA (3, 10, 15 nmol) dose-dependently attenuated the central antinociception of EM-1, *F*_4,33_ = 39.300, *P*<0.001, the EC_50_ value (and 95% confidence limits) is 6.27 (4.90–8.03) nmol (Fig. 4B). Furthermore, 15 nmol RF9 (i.c.v.) fully blocked the EM-1-modulating actions of NPVF and dNPA in the mouse tail-flick test, *F*_3,26_ = 71.266, *P*<0.001; *F*_3,26_ = 51.288, *P*<0.001, respectively (Fig. 4C).

**Figure 5:**
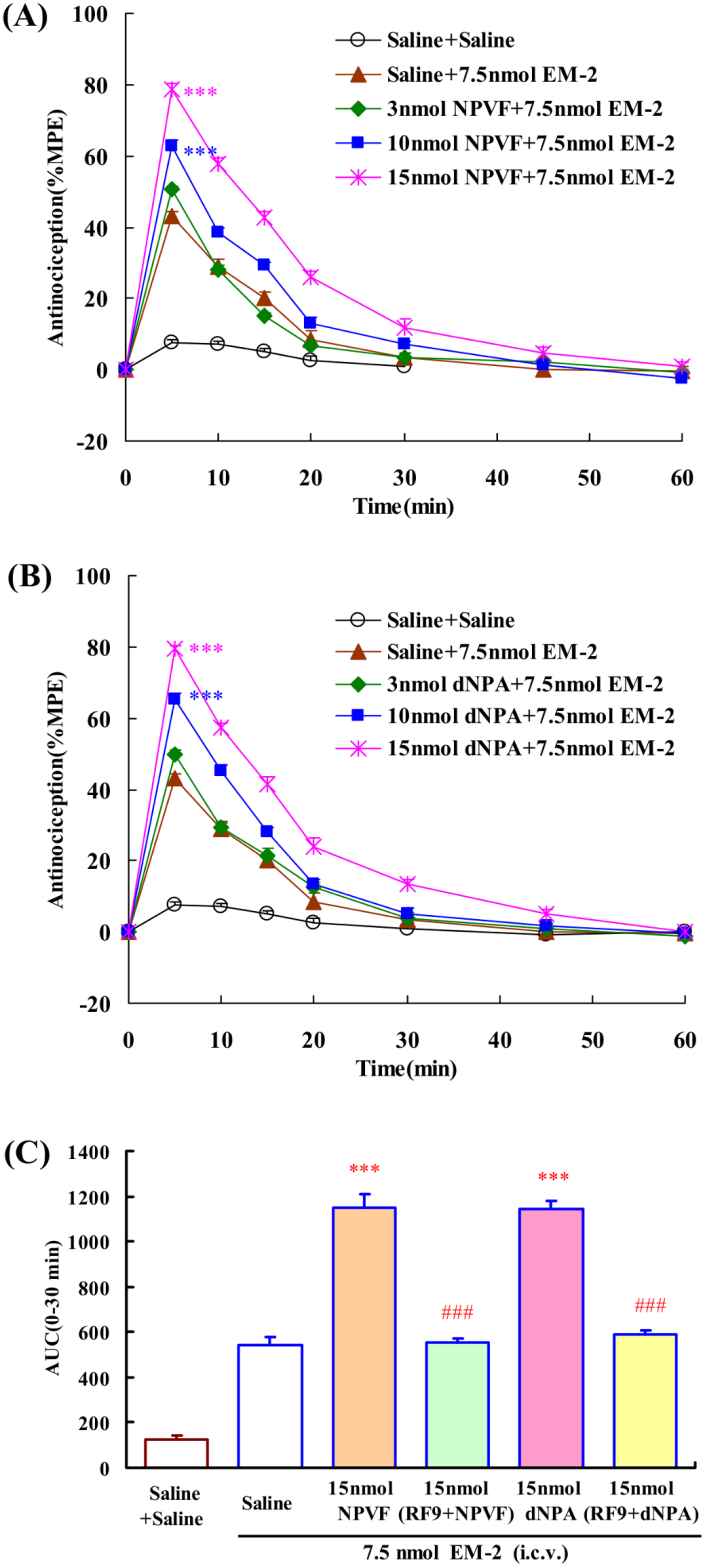
Dose-related effects of i.c.v. administration of NPFF receptor selective agonists on the EM-2 (i.c.v.) induced central antinociception in mouse tail-flick assays. (A) NPVF (3, 10 and 15 nmol) and (B) dNPA (3, 10 and 15 nmol) potentiated EM-1(7.5 nmol) induced central antinociception. (C) Co-administration of RF9 (15 nmol, i.c.v.) antagonized the effects of NPFF (15 nmol, i.c.v.) on EM-2-induced central antinociception in mouse tail-flick test. Each value represents mean ± S.E.M. (n = 7–8 mice/group). ***P<0.001 indicating significant differences compared to Saline + EM-2-injected group. ^###^P<0.001 indicating significant differences from the modulatory effects of NPFF-related peptides in the absence of RF9.

The effects of NPVF and dNPA on EM-2-induced central antinociception were shown in Fig. 5. Both of NPVF (3, 10, 15 nmol) and dNPA (3, 10, 15 nmol) dose-dependently enhanced the central antinociception of EM-2, *F*_4,37_ = 140.108, *P*<0.001 (Fig. 5A); *F*_4,37_ = 151.277, *P*<0.001 (Fig. 5B), respectively. The EC_50_ values (and 95% confidence limits) are 12.58 (11.17–14.18) nmol and 11.82 (10.62–13.18) nmol, respectively. 15 nmol RF9 (i.c.v.) fully blocked the EM-2-modulating actions of NPVF and dNPA in the mouse tail-flick test, *F*_3,30_ = 166.920, *P*<0.001; *F*_3,30_ = 153.518, *P*<0.001, respectively (Fig. 5C).

### Effects of i.c.v. administration of NPFF and related peptides on the central antinociception produced by DAMGO

The previous reports indicated that the central antinociceptive effect induced by both EM-1 and DAMGO (the µ-opioid receptor selective agonist) was selectively mediated by µ-opioid receptor. Thus, DAMGO was used to confirm the modulatory effects of NPFF on µ-opioid receptor mediated central antinociception.

**Figure 6:**
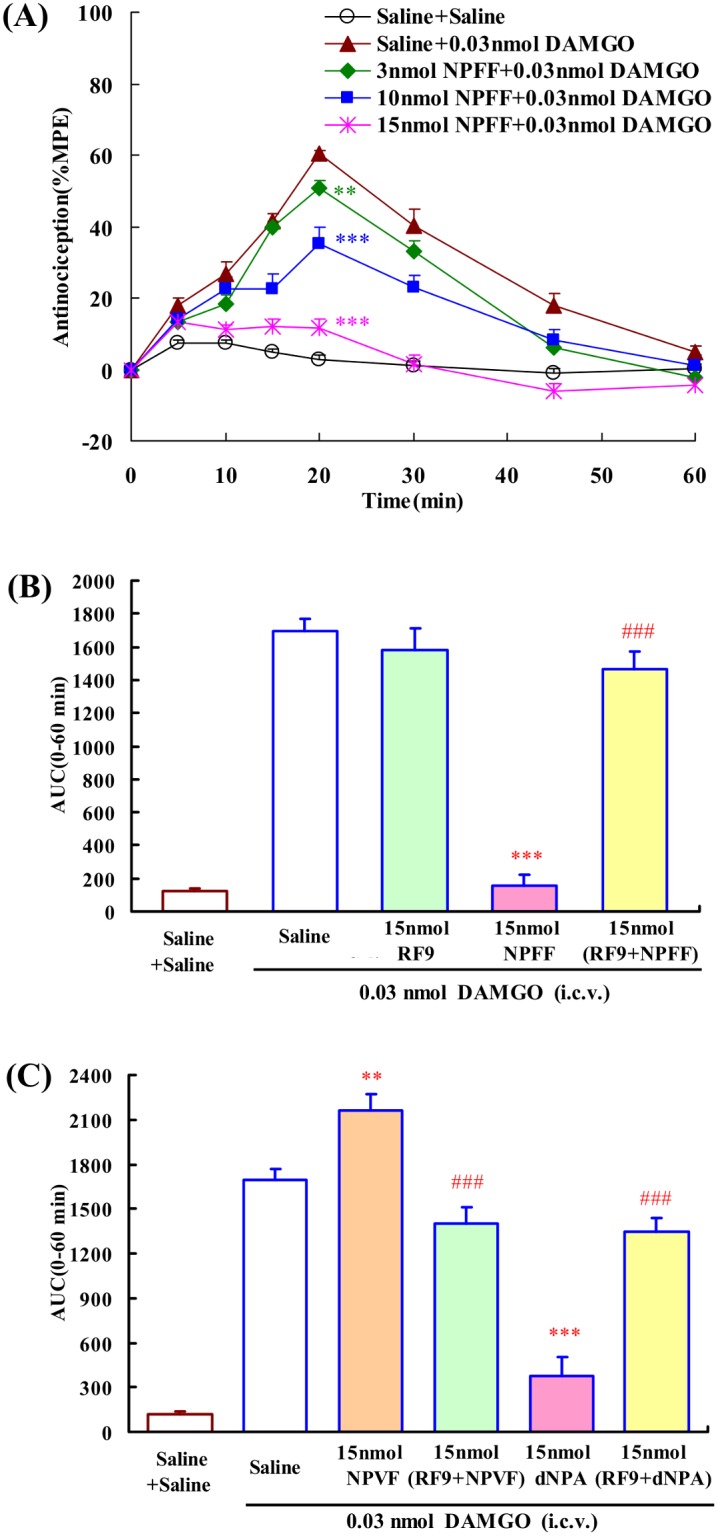
The effects of i.c.v. administration of NPFF and related pepetides on the central antinociception of DAMGO (i.c.v.) in mouse tail-flick test. (A) Dose-related effects of NPFF (3, 10 and 15 nmol) on the central antinociception of DAMGO. (B) Co-administrated RF9 (15 nmol, i.c.v.) antagonized the modulatory effects of NPFF (15 nmol, i.c.v.) on the central antinociception of DAMGO in mouse tail-flick test. (C) The effects of NPVF and dNPA on the central antinociception of DAMGO. Each value represents mean ± S.E.M. (n = 7–8 mice/group). **P<0.01 and ***P<0.001 indicating significant differences compared to Saline + DAMGO-injected group; ^###^P<0.001 indicating significant differences from the modulatory effects of NPFF in the absence of RF9.

Intracerebroventricular administration of 0.03 nmol DAMGO was selected to induce 60% analgesia at the peak effect (Fig. 6). Lateral ventricle administration of NPFF (3, 10, 15 nmol) dose-dependently attenuated the central antinociception of DAMGO with an EC_50_ value (and 95% confidence limits) of 8.19 (6.23–10.76) nmol, *F*_4,32_ = 66.027, *P*<0.001 (Fig. 6A). In addition, co-injected with 15 nmol RF9 (i.c.v.) completely blocked the modulating activity of NPFF on DAMGO-induced central antinociception, *F*_4,31_ = 95.663, *P*<0.001. RF9 itself had no effect on the central antinociception of DAMGO (Fig. 6B).

Intracerebroventricular administration of NPVF (15 nmol) significantly enhanced the central antinociception of DAMGO, (*P*<0.01). In contrast, i.c.v. administration of dNPA (15 nmol) significantly attenuated the central antinociception of DAMGO, (*P*<0.001). Furthermore, 15 nmol RF9 (i.c.v.) fully blocked the DAMGO-modulating actions of NPVF and dNPA in the mouse tail-flick test, *F*_3,26_ = 88.746, *P*<0.001; *F*_3,26_ = 63.138, *P*<0.001, respectively (Fig. 6C).

### Effects of i.c.v. administration of NPFF and related peptides on the central antinociception produced by U69593

Unlike EM-1 and other µ-opioid receptor agonists (including DAMGO), EM-2 can indirectly activate the endogenous κ opioid system [33], [34], [39]. Due to the opposite effects of NPFF on EM-1 and EM-2-induced central antinociception, U69593, a κ-opioid receptor selective agonist, was further used to investigate the role of NPFF on κ-opioid system in the EM-2-modulating action of NPFF.

**Figure 7:**
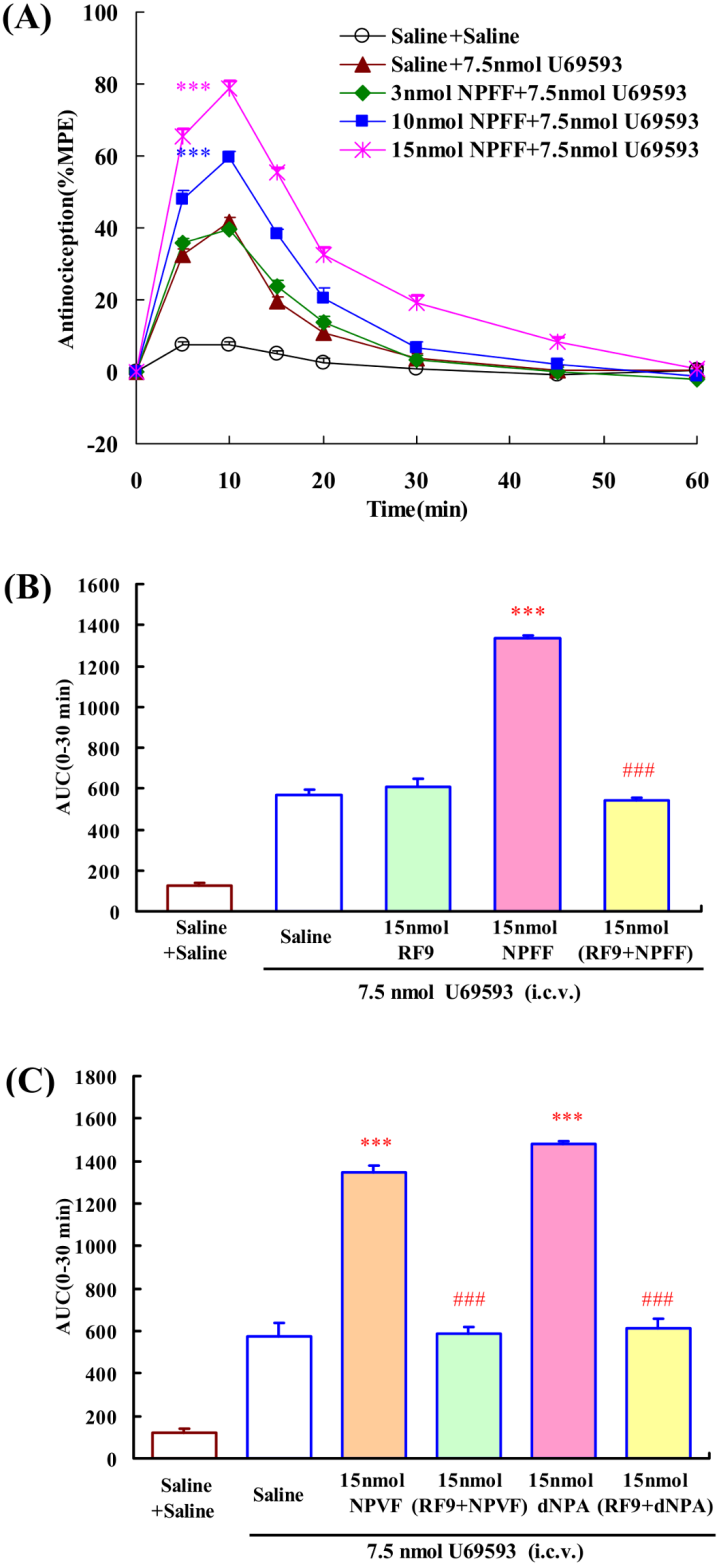
The effects of i.c.v. administration of NPFF and related peptides on the central antinociception of U69593 (i.c.v.) in mouse tail-flick test. (A) Dose-related effects of NPFF (3, 10 and 15 nmol) on the central antinociception of U69593. (B) Co-administrated RF9 (15 nmol, i.c.v.) antagonized the modulatory effects of NPFF (15 nmol, i.c.v.) on the central antinociception of U69593 (i.c.v.). (C) The effects of NPVF and dNPA on the central antinociception of U69593. Each value represents mean ± S.E.M. (n = 7–8 mice/group). ***P<0.001 indicating significant differences compared to Saline + U69593-injected group; ^###^P<0.001 indicating significant differences from the modulatory effects of NPFF in the absence of RF9.

Intracerebroventricular administration of 7.5 nmol U69593 induced 42% analgesia at the peak effect, allowing investigation of both potentiation and reversion of the central antinociception (Fig. 7). Lateral ventricle administration of NPFF (3, 10, 15 nmol) dose-dependently enhanced the central antinociception of U69593 with an EC_50_ value (and 95% confidence limits) of 12.70 (12.05–13.37) nmol, *F*_4,34_ = 277.555, *P*<0.001 (Fig. 7A). In addition, 15 nmol RF9 (i.c.v.) completely blocked the modulating activities of NPFF on U69593-induced central antinociception, *F*_3,27_ = 733.268, *P*<0.001. RF9 itself had no effect on the central antinociception of U69593 (Fig. 7B).

The effects of NPVF and dNPA on U69593-induced central antinociception were shown in Fig. 7C. Both of NPVF (15 nmol) and dNPA (15 nmol) significantly enhanced the central antinociception of U69593 (*P*<0.001). RF9 (15 nmol i.c.v.) fully blocked the U69593-modulating actions of NPVF and dNPA in the mouse tail-flick test, *F*_3,25_ = 130.450, *P*<0.001; *F*_3,27_ = 174.542, *P*<0.001, respectively.

## Discussion

NPFF is widely considered as an opioid-modulating peptide [11], [14]. A great deal of evidence has shown that NPFF and related analogs played important roles in the regulation of opioid-induced analgesia [13], [23]. Previous studies mainly focused on the modulating effects of NPFF on morphine-induced analgesia. But unlike morphine, the endogenous µ-opioid receptor ligands endomorphins, especially EM-2 were reported to induce antinociceptive effect mediated by the different mechanisms [32]–[34], [39], [40]. Thus, the present study was conducted to investigate the modulating role of supraspinal NPFF system on endomorphins-induced antinociception in the mouse tail-flick assay.

Initially, our results showed that i.c.v. injection of EM-1 and EM-2 caused significant antinociception. The central antinociceptive effect of EM-1 was blocked by pretreatment with µ-opioid receptor antagonist β-FNA, but not by κ-opioid receptor antagonist nor-BNI or δ-opioid receptor antagonist naltrindole. In contrast, pretreatment with β-FNA completely blocked the EM-2-induced central antinociception, and nor-BNI partially blocked the EM-2-induced central antinociception. These data indicated that EM-1 induced central antinociception via µ-opioid receptor while the central antinociception of EM-2 was mediated by both µ- and κ-opioid receptors. Indeed the previous studies have revealed that EM-2 induced antinociception via different mechanism compared to other µ-opioid receptor agonists. Tseng et al. reported that pretreatment of mice with an antiserum against dynorphin A(1–17) attenuated the antinociception of EM-2 [34]. Treatment with EM-2 could increace the immunoreactive of dynorphin A(1–17) in spinal perfusates [41]. In addition, the release of dynorphin A(1–17) induced by spinal or supraspinal treatment with EM-2 was demonstrated to mediated by activation of µ-opioid receptor [42], [43]. Taken together, EM-2 initially stimulated µ-opioid receptor, which subsequently induced the release of dynorphin A(1–17) that act on κ-opioid receptor to produce antinociception.

The results in present study demonstrated that i.c.v. administration of NPFF dose-dependently reduced EM-1-induced central antinociception, which was markedly antagonized by the NPFF receptors selective antagonist RF9. These data indicated that the inhibitory effects of NPFF on the central antinociception of EM-1 are mainly mediated by activating NPFF receptors. In contrast, i.c.v. administration of NPFF evoked a marked increase of EM-2-induced central antinociception, and its modulatory effect was also related to activation of NPFF receptors. These data indicated that NPFF exerts opposite effects on central antinociception of EM-1 and EM-2.

NPFF is a non-selective agonist of NPFF receptors. Thus, the selective agonists for NPFF_1_ and NPFF_2_ receptors, NPVF and dNPA, respectively [8]–[10] were used to further explore the roles of two NPFF receptor subtypes in the endomorphins-modulating effects.

In the present studies, our results demonstrated that the selective agonists of NPFF receptors had opposite modulating effects on EM-1-induced central antinociception. NPVF enhanced the antinociception of EM-1 while dNPA attenuated EM-1-induced antinociception at supraspinal level. In addition, the NPFF receptors antagonist RF9 significantly reduced the EM-1-modulating activities of NPVF and dNPA, indicating that the activations of central NPFF_1_ and NPFF_2_ receptors caused opposite effects on central antinociception induced by EM-1, and the EM-1-modulating activity of NPFF was mainly mediated by NPFF_2_ receptor.

Similarly to NPFF, administration of NPVF and dNPA by supraspinal route enhanced the central antinociception of EM-2. In addition, the NPFF receptors antagonist RF9 significantly prevented the EM-2-modulating activities of these two selective agonists, indicating that the activations of central NPFF_1_ and NPFF_2_ receptor caused the similar increase in central antinociception induced by EM-2, and EM-2-modulating activity of NPFF is mediated by both of NPFF_1_ and NPFF_2_ receptors.

In the present work, the results demonstrated that NPFF exerted opposite effects on central antinociception of EM-1 and EM-2. It is notable that EM-1-induced central antinociceptive effect was mediated by µ-opioid receptor, while the central antinociception of EM-2 was mediated by both µ- and κ-opioid receptors. Thus, it is possible that the opposite modulatory effects of NPFF on endomorphins-induced central antinociception resulted from the different mechanisms of endomorphins-induced nociceptive modulation. Furthermore, the µ- and κ-opioid receptor selective agonist DAMGO and U69593, respectively, were used to investigate the roles of µ- and κ-opioid system in modulatory effects of NPFF on endomorphins-induced central antinociception.

Our results showed that i.c.v. injected NPFF could attenuate the central antinociception induced by DAMGO. NPVF enhanced but dNPA attenuated DAMGO-induced central antinociception. In addition, the NPFF receptors antagonist RF9 significantly reduced the DAMGO-modulating activities of NPFF and related pepetides. These results indicated that NPFF exactly modulated EM-1- and DAMGO-induced central antinociception in the same manner. Moreover, the previous studies have shown that NPFF system induced different modulating effects on morphine analgesia. NPFF was reported to attenuate the analgesia induced by morphine in the mouse tail-flick assay [24]. NPFF stable analog 1DMe also reduced the central antinociception of morphine and DAMGO in mice [25], [44]. NPFF_1_ receptor selective agonist NPVF enhanced but NPFF_2_ receptor selective agonist dNPA attenuated morphine-induced central antinociception [10], [44]. Taken together, NPFF has similar inhibitory effects on EM-1, morphine and DAMGO, which further supports an anti-opioid character of NPFF system.

It is interesting that NPFF could enhance EM-2-induced central antinociception at supraspinal level. EM-2 induced the antinociception via the mechanism different from that of DAMGO or EM-1, and in part by the stimulation of κ-opioid receptor [34], [39]. Thus, we hypothesized that the enhancement of NPFF in EM-2-induced antinociception might result from the interaction between NPFF and κ-opioid systems. In fact, the present results revealed that i.c.v. administration of NPFF enhanced the central antinociception of U69593 via NPFF receptors. The results using NPVF and dNPA showed that both NPFF_1_ and NPFF_2_ receptors participant in the potentiation of NPFF on U69593-induced central antinociception. The same pattern in modulatory effects of NPFF on central antinociception produced by EM-2 and U69593 confirmed that κ-opioid system was participant in the enhancement of NPFF on EM-2-induced central antinociception.

Central administration of EM-2 activated µ-opioid receptor which induced releases of dynorphins that acted on κ-opioid receptor to induce antinociception [32]–[34]. However, NPFF inhibited µ-opioid-induced central antinociception, but enhanced κ-opioid-induced central antinociception. Accordingly, in theory, if NPFF has modulating effect on both µ-opioid and κ-opioid receptors, NPFF would attenuate µ-opioid agonism and enhance κ-opioid agonism, leading to a biphasic modulation in EM-2-induced antinociception. It seems difficult to explain why NPFF only exerted pro-opioid action and enhanced EM-2-induced central antinociception. However, basing on the present findings, we suppose that NPFF might not block the dynorphins release induced by EM-2. Results in Fig. 7 have shown that NPFF could enhance κ-opioid induced central antinociception. So the interaction between NPFF and κ-opioid system could be an explanation that NPFF enhanced EM-2-induced central antinociception. However, further pharmacological studies are required to prove the detailed mechanism involved in the interaction between NPFF and EM-2.

Our recent study has shown that NPFF system inhibited the acquisition and expression of EM-2-induced conditioned place aversion [45]. However, in the present study, our results have shown that NPFF system enhanced EM-2-induced central antinociception. Thus, supraspinal NPFF system exerted different modulating roles in rewarding and nociceptive effects of EM-2. In fact, the different modulating effects in the different models have been found in other studies. For instance, the mGlu5 receptor antagonist 3-[(2-methyl-1,3-thiazol-4-yl) ethynyl]pyridine (MTEP) attenuated operant self-administration of morphine [46], but potentiated morphine antinociception [47], [48]. In addition, in theory, if the strategy using combination treatment of EM-2 and NPFF is applied in pain management, the antinociception of EM-2 would be enhanced by NPFF, and EM-2-induced conditioned place aversion would be inhibited. It would pave a way for the development of a new strategy for powerful analgesia with lower side effects.

In conclusion, the body of data derived from our present experiments proves, for the first time, NPFF has opposite effects on central antinociception induced by EM-1 and EM-2 via complex mechanisms. In addition, the enhancement of NPFF on EM-2-induced antinociception may be related to the modulating effect of NPFF on κ-opioid receptor at the supraspinal level. Furthermore, the EM-1-modulating activity of NPFF is mainly mediated by NPFF_2_ receptor while the EM-2-modulating activity of NPFF is mediated by both NPFF_1_ and NPFF_2_ receptors. Moreover, our study should be helpful to better understand the pharmacological function of NPFF and its relationship with the endogenous opioid system.
